# Real-world experience of water vapour therapy (Rezum) in patients with benign prostatic enlargement: a retrospective single-center study

**DOI:** 10.1038/s41391-024-00836-w

**Published:** 2024-04-24

**Authors:** Mathias Wolters, Martin Krastel, Thorben Winkler, Hamza Idais, Mehrdad Mazdak, Hossein Tezval, Markus A. Kuczyk, Christoph-A. J. von Klot

**Affiliations:** 1 Department of Urology and Urological Oncology, Hannover Medical School (MHH), Carl-Neuberg-Str.1, 30625 Hannover, Germany; 2 Department of Urology, DIAKO Hospital Flensburg, Knuthstraße 1, 24939 Flensburg, Germany; 3 Department of Urology, St. Bernward Hospital Hildesheim, Treibestraße 9, 31134 Hildesheim, Germany

**Keywords:** Medical research, Prostatic diseases

## Abstract

**Background:**

Water vapor thermal therapy (Rezum) is a minimally invasive treatment for benign prostatic enlargement (BPE). Studies reporting urodynamic results regarding the procedure are rare. Our study aimed to assess the effectiveness of Rezum on urinary outcome parameters in a consecutive series of patients and compare urodynamic data before and after treatment.

**Methods:**

We retrospectively evaluated all the patients treated with Rezum between 07/2017 and 12/2023 at our institution. Patients who had more than one Rezum intervention, those who were unable to void (i.e., catheter-dependent patients), and those with insufficient data were excluded from the data analysis. Descriptive outcomes, such as symptom scores (IPSS, IPSS-QoL), peak flow in uroflowmetry (Qmax), post-micturition residual urine volume (PVR), and prostate volume (PVol), were analyzed. If available, preoperative and postoperative urodynamic results were evaluated.

**Results:**

In total, 250 Rezum procedures were performed during the observational period. After applying the exclusion criteria, the data from 193 patients were included in the analysis. Patients achieved significant symptom relief as measured using the IPSS (46% reduction) and IPSS-QoL scores (41% reduction). Qmax improved by 4.8 ml/s, as the mean PVR significantly decreased by 50%. PVol and PSA values decreased by 30% and 27.5%, respectively. In 19/193 patients with a urodynamic evaluation, pre- and postoperative data analysis showed a significant reduction in the bladder outlet obstruction index (BOOI) by approximately 70%.

**Conclusions:**

Rezum is effective and can improve urinary symptoms. In appropriate patients, Rezum can significantly reduce the bladder outlet obstruction (BOO).

## Introduction

According to current guidelines, transurethral resection of the prostate (TURP) is the standard of care for moderate-to-severe drug-refractory lower urinary tract symptoms (LUTS) in patients with prostate volumes up to 80 cc [1, 2].

Several minimally invasive treatment options for BPE have been introduced in recent decades, including the Rezum System (Boston Scientific, Marlborough, MA, US) [3, 4], which was approved by the United States Food and Drug Administration (US FDA) in 2015 (510(k) number K150786). Rezum involves injecting radio-frequency-generated convective water vapor thermal energy into the prostatic tissue under cystoscopic control with a retractable needle. Water vapor is delivered for 9 s at different overlapping treatment sites in the prostatic urethra, causing immediate cell necrosis which result in improved LUTS. The maximum effect of therapy is expected to be 6 weeks to 3 months postoperatively. In contrast to other minimally invasive therapies (e.g., Urolift), Rezum can be applied to patients with a median prostate lobe.

In 2021, a randomized controlled clinical trial reported significant improvements in patient-related symptom relief, quality of life, and uroflowmetry measurements over a 5-year follow-up period [5]. Rezum has also been shown to be safe and effective in multiple retrospective studies [6–11], but data on its effect on bladder outlet obstruction (BOO) are rare [12].

It is important to mention that BOO can only be diagnosed using pressure-flow measurements. BOO is defined by increased detrusor pressure in combination with decreased urinary flow, and threshold values to distinguish between non-obstructed and obstructed bladders have been established. Various formulas (e.g., BOOI) and nomograms (e.g., ICS, Schäfer nomogram, or CHESS-nomogram) facilitate the assessment of BOO in individual patients [13–17].

Our study aimed to evaluate the clinical outcomes of Rezum treatment in a consecutive series of patients and provide further evidence of its efficacy. Therefore, urodynamic data before and after treatment were compared to better understand the effect of Rezum on BOO.

## Methods

### Study population

Patients aged >40 years with symptomatic BPE without prior surgical intervention of the prostate who were treated with Rezum between 07/2017 and 12/2023 were included and retrospectively analyzed. Patients who underwent more than one Rezum intervention and those who were unable to void prior to Rezum therapy (i.e., urinary retention, continuous transurethral, suprapubic, or intermittent catheterization) were excluded from data analysis. Only patients with sufficient data before and after the treatment were included.

Further exclusion criteria were prostate cancer, urethral strictures, prostatitis, active urinary tract infection, and neurogenic bladder disorders.

This study was conducted in accordance with the current standard of care according to the recommendations of the European Association of Urology (EAU) guidelines on the management of non-neurogenic male lower urinary tract symptoms (LUTS), including benign prostatic obstruction (BPO) [1]. The institutional Ethics Committee approved this retrospective evaluation of the anonymized clinical data (reference number: 10234_BO_K_2022). All participants provided written informed consent in accordance with the Declaration of Helsinki.

### Clinical assessment

The following data, patient characteristics, and interventional data were used for analysis: age, prostate specific antigen value (PSA), PVol (measured using transrectal sonography of the prostate (TRUS)), duration of procedure (min), blader neck-colliculus-distance (BCD, cm), number of injections subdivided in the left, right, and median lobes, hospitalization time (days), and discharge with a catheter after postoperative urinary retention.

Before treatment, patients completed the validated German International Prostate Symptom Score (IPSS) and IPSS-Quality of Life (QoL) scores. To evaluate symptom severity and treatment results, the IPSS was subdivided into the IPSS voiding subscore (IPSS-V) and the IPSS storage subscore (IPSS-S). The IPSS-V is the sum of the answers to questions 1 (incomplete emptying), 3 (intermittency), 5 (weak stream), and 6 (strain-to-void). In contrast, the IPSS-S is the sum of the answers to questions 2 (frequency), 4 (urgency), and 7 (nocturia).

To perform free uroflowmetry, all the patients were asked to void with a full bladder. Only measurements >125 ml were considered suitable for the analysis. PVR was evaluated using transabdominal ultrasonography of the bladder. All sonographic examinations were performed by an experienced physician and documented in the medical records. Follow-up assessments were conducted after six weeks, as well as three, six and twelve months after treatment voluntarily. Device- or procedure-related adverse events were assessed, and complications were recorded using the Clavien-Dindo classification system [18].

### Urodynamic assessment

Indications for preoperative urodynamic measurements were applied according to the EAU guidelines for BPE/LUTS [1]. Data on urodynamic measurements were retrospectively analyzed to identify patients in whom urodynamic evaluation was performed pre- and postoperatively. Urodynamic investigations were conducted by experienced physicians following the Good Urodynamic Practices Standards suggested by the International Continence Society [19]. The urodynamic investigations in our clinic were performed in a standardized manner, as reported by Oelke et al. [17]. First, free uroflowmetry was performed, and PVR was measured using transurethral catheterization immediately after voiding and before starting urodynamic evaluation. Therefore, a 6 French (Fr) double-lumen catheter was placed in the bladder to quantify PVR volume, after which the bladder was filled and the intravesical pressure (pVes) was measured. To assess intra-abdominal pressure (Pabd), a 10 Fr single-lumen catheter was positioned in the rectum, and both water-filled catheters were connected with external pressure transducers at the level of the pubic symphysis. The patient was placed in a sitting position, and the bladder was filled with sterile physiological saline solution at a temperature of 37 °C and a speed of 25–50 ml/min until the patient reported a strong urge to void. The patient then voided in the sitting or standing position according to his normal habits, and pressure flow measurement was performed. Cystometry and pressure flow measurements were performed at least twice during the same urodynamic examination, to ensure accurate and reliable results.

### Urodynamic parameters for analysis

The following parameters of free uroflowmetry (Qmax, voided volume, PVR, bladder capacity, voiding efficiency) and pressure-flow measurements (filling sensations, detrusor overactivity, compliance, detrusor pressure at maximum flow (Pdet Qmax)), maximum detrusor pressure, Qax, BOOI, bladder contractility index (BCI; PdetQmax + 5Qmax), and detrusor contractility (Wmax) were recorded.

Because free uroflowmetry, PVR, cystometry, and pressure-flow examinations were performed at least twice, only representative recordings were used for the analysis. Free uroflowmetry with the highest Qmax value was selected, and the corresponding PVR measurements were used. Bladder capacity on uroflowmetry was calculated by adding the voided volume and PVR. To determine the percentage of bladder emptying in relation to bladder filling, voiding efficiency (VE) was calculated using the following formula: VE = (voided volume/bladder capacity) × 100 [%]. BOOI was used to determine BOO grade. BOOI was calculated by the formula: BOOI = P_detQmax_-2Q_max_ [cm H_2_O].

The ICS BOO nomogram was used to calculate the BOOI by plating Qmax against pdet@Qmax. Based on the nomogram, patients were categorized as being obstructed, unobstructed, or equivocal. The nomogram was calculated manually using the formula BOOI=pdet@Qmax-(2xQmax). A BOOI < 20 is considered non-obstructed, a BOOI between 20 and 40 as equivocal, and a value of >40 as obstructed.

### Operative procedure

The Rezum system was used following the manufacturer’s recommendations for the treatment of both prostatic lobes as well as the central zone or median lobe, as previously described [20, 21]. All interventions were performed under light or general anesthesia, but none were performed under local anesthesia. Postoperatively, all patients received an 18 Fr transurethral indwelling catheter that was removed on the second postoperative day. If patients were unable to void due to initial swelling of the prostatic urethra, a transurethral or suprapubic catheter was placed and the patients were discharged with the catheter. In these patients, it was recommended that a trial without a catheter be conducted no earlier than one week postoperatively in an outpatient setting.

### Statistical analysis

Patient data were stored in our institutional database, comprising relational data in SQLite csv-format. We used a web-based relational database with an internally created RShiny-based API for data storage and analysis [22, 23]. Data selection, manipulation, aggregation, and filtering of time-dependent data were performed using R’s dplyr-package [24].

Statistical evaluations and illustrations were performed using R Statistical Software (R version 4.1.0, Vienna, Austria, https://www.R-project.org/). For line and scatter plots, we used R’s ggplot2-package [25]. For the line plot depicting changes in quality of life, we used a jitter function to enhance the visualization for each individual patient.

For descriptive data presentation, categorical data are presented as absolute numbers and percentages. Continuous variables are presented as either the mean and standard deviation or the median with range. Differences in clinical data before and after the Rezum procedure were assessed using the *t*-test for numerical data and Fisher’s exact test for categorical data. Statistical significance was set at *p* ≤ 0.05. Pre-intervention data were obtained up to 200 days before the intervention. Post-interventional data were obtained within 50–300 days after the intervention. In cases in which more than one examination date was available, subsequent post-interventional data were selected for analysis. In cases where patients underwent more than one urodynamic evaluation prior to Rezum, we chose urodynamic data with the shortest time interval.

## Results

In total, 250 patients were treated during the observational period. Of these, four patients who underwent more than one Rezum procedure were excluded. Patients with urinary retention (*n* = 53), that is, those requiring intermittent self-catheterization and suprapubic or transurethral catheters, were also excluded from this study. In addition, patients were required to have sufficient clinical data before and after the Rezum procedure, leaving 193 patients for the final data analysis. The median follow-up period was 5.25 months (0.8–50.9, IQR 8.8 months).

The median patient age was 68.0 years (63.0–77.0, IQR 14.0), and the mean PVol at baseline was 56.5 ± 28.5 cc (35.0–70.0, IQR 35.0). A total of 158 patients had a PVol < 80cc (81.9%), and 35 patients had a PVol ≥ 80cc (18.1%). The patient characteristics and interventional data are summarized in Table 1.

**Table 1:** Pre- and perioperative patients’ characteristics and interventional data.

Parameter	Number (%) /mean ± SD	Quartile 25%	Quartile 75%	IQR
**Total patients (*n*)**	193			
**Age (yrs., median)**	68.0	63.0	77.0	14.0
**PSA value (ng/ml)**	3.4 ± 3.2	1.3	4.5	3.2
**IPSS**				
IPSS, score (median)	21.0	17.0	24.0	7.0
IPSS, voiding subscore (median)	11.5	8.2	15.0	6.8
IPSS, storrage subscore (median)	9.0	7.0	12.0	5.0
IPSS, quality of life (median)	4.0	4.0	5.0	1.0
**Prostate volume (cc)**				
Prostate volume total (cc)	56.5 ± 28.5	35.0	70.0	35.0
Prostate volume < 80 cc	158 (81.9%)			
Prostate volume ≥ 80 cc	35 (18.1%)			
**Interventional data**				
Bladder neck-colliculus distance (cm)	3.6 ± 1.4	2.5	4.5	2.0
REZUM left prostate lobe per patient	3.6 ± 1.5	3.0	4.0	1.0
REZUM right prostate lobe per patient	3.5 ± 1.4	2.0	4.0	2.0
REZUM median prostate lobe per patient^*^	1.0 ± 1.3	0.0	2.0	2.0
Duration of procedure (minutes)	7.4 ± 5.2	4.0	9.0	5.0
**Hospitalization (days)**	2.8 ± 1.6	2.0	3.0	1.0
**Urinary retention after Rezum**	21 (10.9%)			
**No urinary retention after Rezum**	172 (89.1%)			

Preoperative and perioperative characteristics and interventional data of 193 patients who underwent the Rezum procedure for benign prostate hyperplasia.

*PSA* prostate prostate-specific antigen, *IPSS* International prostate symptom score.

^*^The median prostate lobe was treated in 82 patients.

All interventions were completed without device- or procedure related adverse events. There were no major complications (Clavien-Dindo score ≥3). 82 patients (42.5%) received treatment of the median prostate lobe. The mean operative time was 7.4 ± 5.2 min. The mean length of hospital stay was 2.8 ± 1.6 days. In total, 172 patients (89.1%) were discharged without a urinary catheter. Twenty-one patients (10.9%) were discharged with a suprapubic or transurethral catheter postoperatively because of a high PVR or urinary retention.

**Fig. 1:**
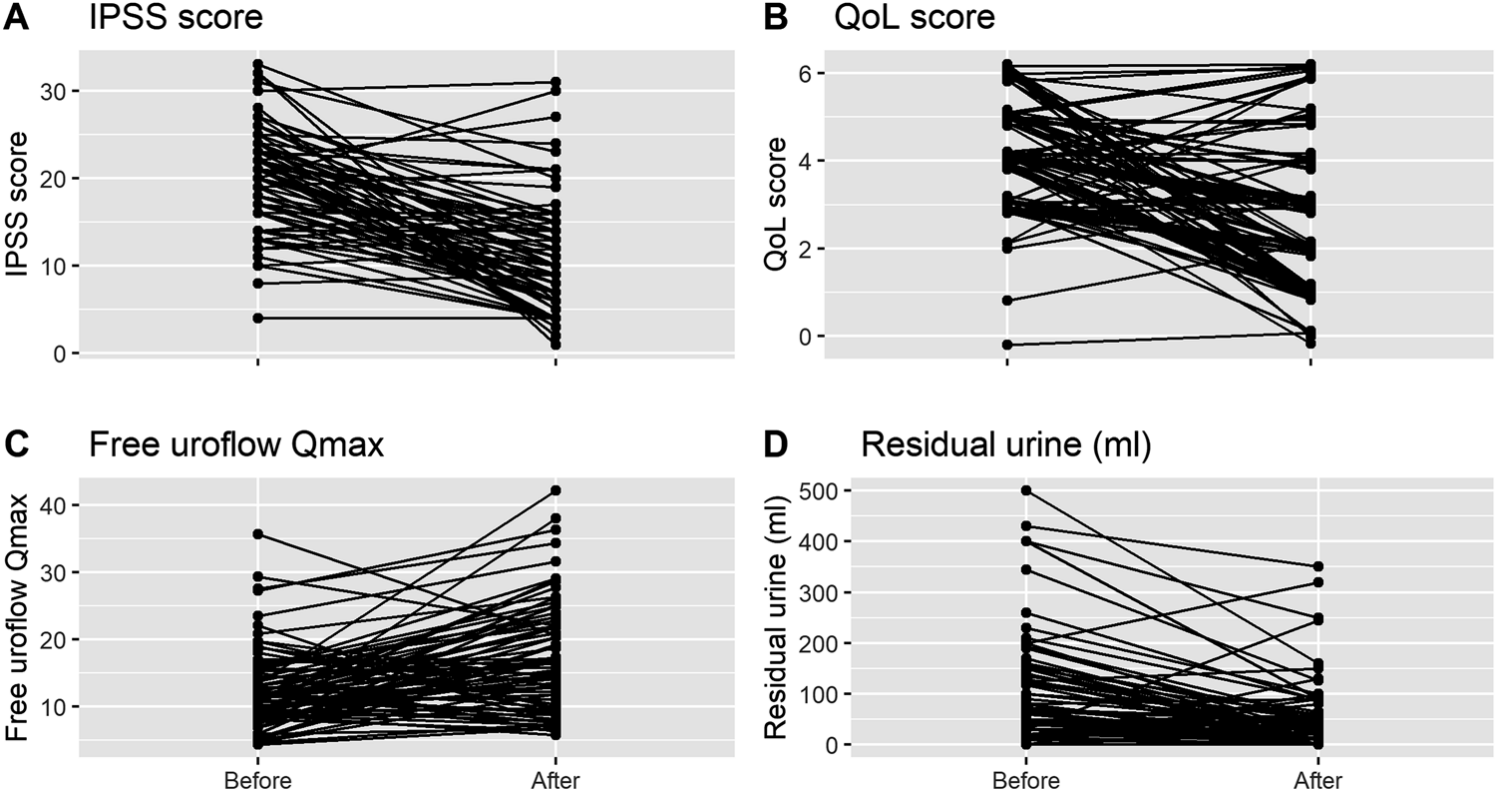
Line graph showing four parameters before and after the Rezum procedure. **A** IPSS (International prostate symptom score (20.3 ± 5.9 vs. 11.0 ± 6.6; *p* < 0.001)), **B** QoL (Quality of life (4.2 ± 1.2 vs. 2.6 ± 1.7; *p* < 0.001)), **C** Qmax (maximum flow, ml/sec (12.5 ± 5.8 vs. 17.3 ± 8.1; *p* < 0.001)) and **D** residual urine (107.0 ± 108.4 vs. 54.1 ± 69.6; *p* < 0.001).

Regarding overall patient-reported outcomes, LUTS improved significantly, as measured by the IPSS and QoL scores. IPSS improved from 20.3 ± 5.9 to 11.0 ± 6.6 (46% reduction, *p* < 0.001, Fig. 1A) and IPSS-QoL from 4.4 ± 1.2 to 2.6 ± 1.7 score (41% reduction, *p* < 0.001, Fig. 1B). Mean Qmax significantly improved by 4.8 ml/s from 12.5 ± 5.8 ml/s to 17.3 ± 8.1 ml/s (38% improvement, *p* < 0.001, Fig. 1C) post-interventionally were as PVR significantly decreased by 49% from 107.0 ± 108.4 ml to 54.1 ± 69.6 ml (*p* < 0.001, Fig. 1D). The voiding efficiency improved by approximately 13%, from 72.7 ± 19.2% to 81.9 ± 16.2%. Figure 1 illustrates the changes in IPSS and QoL scores as well as the free Qmax rates and PVR before and after treatment.

PVol in TRUS significantly decreased by 30% from 57.6 ± 33.5 cc to 40.2 ± 24.9 cc (*p* = 0.001) and PSA value decreased from 3.5 ± 2.9 ng/ml to 2.9 ± 2.4 ng/ml (27.5%; *p* = 0.068). The treatment results are outlined in Table 2.

**Table 2:** Treatment results.

Parameter	Pre-operative	Post-operative	*p*-value	test
**IPSS**				
IPSS, score	20.3 ± 5.9	11.0 ± 6.6	**<0.001**	*t*-test
IPSS, voiding subscore	11.3 ± 4.4	4.6 ± 4.0	**<0.001**	*t*-test
IPSS, storrage subscore	9.0 ± 2.7	6.4 ± 3.7	**<0.001**	*t*-test
IPSS, Quality of life score	4.2 ± 1.2	2.6 ± 1.7	**<0.001**	*t*-test
** Prostate volume, TRUS (cc)**	57.6 ± 33.5	40.2 ± 24.9	**<0.001**	*t*-test
** PSA value (ng/ml)**	3.5 ± 2.9	2.9 ± 2.4	0.068	*t*-test
**Free uroflowmetry**				
Qmax (ml/sec)	12.5 ± 5.8	17.3 ± 8.1	**<0.001**	*t*-test
Voided volume (ml)	248.6 ± 151.8	236.8 ± 161.8	0.547	*t*-test
Residual urine (ml)	107.0 ± 108.4	54.1 ± 69.6	**<0.001**	*t*-test
Bladder capacity (ml)	351.2 ± 205.2	294.8 ± 191.3	**0.024**	*t*-test
Voiding efficiency	72.7 ± 19.2	81.9 ± 16.2	**0.001**	*t*-test
**Multichannel urodynamics**				
First filling sensation (ml)	163.9 ± 101.6	160.5 ± 80.4	0.900	*t*-test
Urge to urinate (ml)	208.5 ± 116.9	230.2 ± 102.9	0.453	*t*-test
Cystometry, bladder capacity (ml)	321.8 ± 126.7	331.6 ± 125.7	0.778	*t*-test
Detrusor pressure at maximum capacity (cmH_2_O)	19.8 ± 18.1	18.7 ± 26.5	0.876	*t*-test
Detrusoroveractivity (Yes)	14 (7.3%)	14 (7.3%)	1.000	Fisher’s
*Detrusoroveractivity (No)	179 (92.7%)	179 (92.7%)	1.000	Fisher’s
Compliance (ml/cm H_2_O)	52.7 ± 71.8	45.9 ± 51.2	0.701	*t*-test
Detrusor pressure at maximum flow (cmH_2_O)	85.3 ± 30.6	47.5 ± 23.4	**<0.001**	*t*-test
Maximum detrusor pressure (cmH_2_O)	100.9 ± 38.5	69.4 ± 30.2	**<0.001**	*t*-test
Qmax (ml/sec)	6.9 ± 2.9	12.8 ± 4.3	**<0.001**	*t*-test
BOOI (cmH_2_O)	70.3 ± 31.8	21.6 ± 27.2	**<0.001**	*t*-test
BCI (Bladder contractility index)	118.9 ± 34.5	110.8 ± 28.7	0.176	*t*-test
Maximum detrusor contractility (W/m^2^)	13.8 ± 7.2	10.9 ± 5.2	**0.042**	*t*-test
Residual urine (ml)	94.4 ± 117.7	45.1 ± 123.4	0.228	*t*-test

Treatment results for 193 patients comparing preoperative and postoperative clinical parameters. Patients undergoing the Rezum procedure for benign prostate hyperplasia.

Significant differences are bolded.

*PSA* prostate prostate-specific antigen, *IPSS* international prostate symptom score, *Qmax* maximum free flow, *BOOI* bladder outlet obstruction index, *BCI* bladder contractility index.

Regarding the subgroup of patients with larger prostates ( ≥80 cc), PVol significantly decreased by 32% from 112.6 ± 29.1 cc to 76.6 ± 27.2 cc (*p* < 0.001) were as the change in PSA level was not significant with a decrease from 5.3 ± 3.3 ng/ml to 4.1 ± 1.5 ng/ml (23%; *p* = 0.398).

Although in this group IPSS significantly improved from 18.6 ± 8.9 to 11.2 ± 6.6 (40% reduction, *p* < 0.001) improvements in IPSS-QoL score from 4.3 ± 1.3 to 3.0 ± 1.7 (30% reduction, *p* < 0.040) and mean Qmax by 3.0 ml/s from 14.4 ± 7.0 ml/s to 17.4 ± 9.2 ml/s (21% improvement, *p* < 0.123) were not as pronounced.

While preoperative urodynamic evaluation was performed according to the current guidelines, postoperative urodynamic assessment was carried out in patients who continued to have storage symptoms after undergoing Rezum in most cases. Focusing on the subgroup of 19 patients with urodynamic evaluation pre- and postoperatively, significant differences were noted in detrusor pressure at maximum flow (decrease from 85.3 ± 30.6 cmH_2_O to 47.5 ± 23.4 cmH_2_O, *p* < 0.001), maximal detrusor pressure (decrease from 100.9 ± 38.5 cmH_2_O to 69.4 ± 30.2 cmH_2_O, *p* < 0.001), Qmax during the pressure flow study (increase from 6.9 ± 2.9 ml/s to 12.8 ± 4.3 ml/s, *p* < 0.001), and BOOI (from 70.3 ± 31.8 cmH_2_O to 21.6 ± 27.2 cmH_2_O, *p* < 0.001). As expected, there was also an effect on bladder contractility (decrease from 13.8 ± 7.2 to 10.9 ± 5.2; *p* < 0.042).

**Fig. 2:**
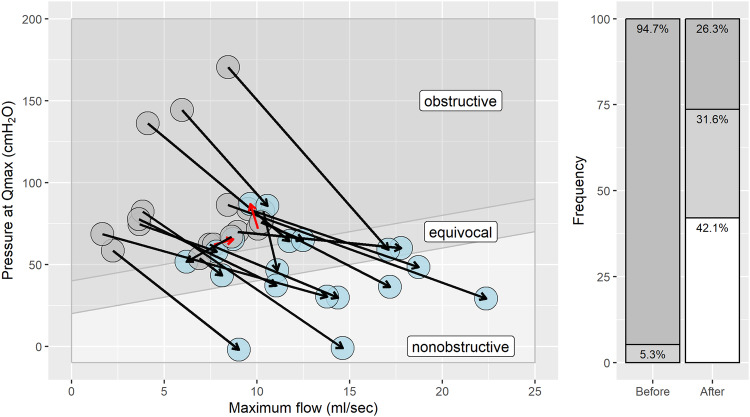
Scatterplot of maximum flow (Qmax [ml/sec]) vs. intravesical pressure at Qmax [cmH_2_O] during the urodynamic pressure flow study. The calculated bladder outlet obstruction index (BOOI) improved after Rezum (70.3 ± 31.8 vs. 21.6 ± 27.2; *p* < 0.001). Measurements before Rezum (grey) and after Rezum (blue) show a clear improvement in most patients (black arrow), with only two patients showing worsening of symptoms (red arrows). The bar plot shows the percentages of patients with obstructive (dark grey), equivocal (light grey), and non-obstructive (white) ICS classification before and after Rezum treatment.

In addition, we used the ICS BOO nomogram to illustrate BOO before and after treatment. Eighteen patients (94.7%) with urodynamic evaluations were classified as obstructed and one patient (5.3%) as non-obstructed. After Rezum treatment, eight patients (42.1%) were classified as unobstructed and six patients (31.6%) as equivocal or obstructed (26.3%). Figure 2 shows the BOOI values of the patients before and after treatment, and the percentage of patients classified into different BOO grades according to the ICS BOO classification.

In patients with urodynamic study the total IPSS score (20.9 ± 5.7 vs. 13.8 ± 5.7 (*p* = 0.006)) and the IPSS voiding subscore (10.9 ± 4.2 vs. 5.3 ± 4.0 (*p* = 0.006)) significantly improved but the IPSS storage subscore (10.0 ± 3.3 vs. 8.5 ± 3.2 (*p* = 0.185)) and the QoL score (3.5 ± 1.5 vs. 3.3 ± 1.8 (*p* = 0.746)) did not show a significant improvement. As in the entirety of patients we noted a significant change in prostate volume in patients with urodynamic assessment comparing pre- and postoperative data (45.0 cc ± 16.6 cc vs. 29.6 cc ± 14.5 cc (*p* = 0.003)).

**Fig. 3:**
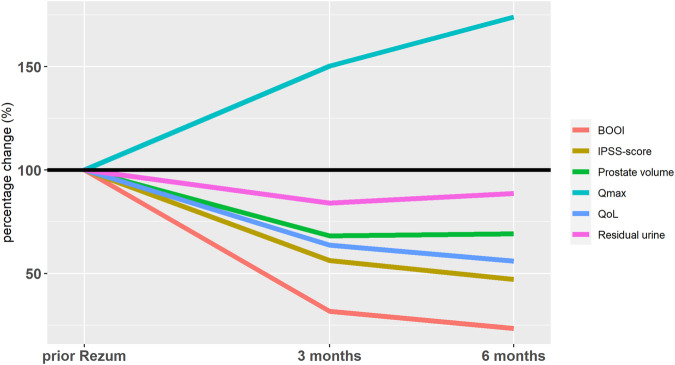
Follow-up for IPSS score, Qmax, PVol, IPSS-QoL, PVR and BOOI. Depicted is the mean change of percentage from baseline prior to Rezum.

Figure 3 offers a concise overview of the temporal progression observed during the follow-up regarding IPSS, IPSS-Qol, Qmax, PVol, PVR and BOOI.

## Discussion

This study aimed to assess the effectiveness of Rezum therapy in a consecutive series of patients. Our current data represent one of the largest retrospective studies so far of patients treated with Rezum in Germany and to the best of our knowledge this is the second study that provides detailed urodynamic data before and after treatment. Overall, Rezum appears to be a promising option for LUTS treatment using a minimally invasive approach.

In our study, we confirmed the early clinical outcomes of effectiveness and safety in line with a previously published prospective randomized control trial [5], some retrospective series [6–8, 26], and two prospective studies [27, 28].

Patient-reported outcomes, as reflected in IPSS and IPSS-QoL score improvements of 46% and 41%, respectively, correspond to the observations of McVary et al. [5] (46.7% reduction in IPSS and 42.9% reduction in IPSS-QoL) and Darson et al. (54.2% reduction in the IPSS) [6].

In the subgroup of patients with larger prostate volumes, our data suggest that the therapeutic effect appears to be poorer since only the improvement of the IPSS score was significant, but the improvement in IPSS-QoL was just barely significant.

Reflecting changes in uroflowmetry, our findings outline an enhancement in Qmax of 38%, which seems appropriate compared with the 49.5% improvement demonstrated by McVary et al. and the 51.4% improvement reported by Darson et al. Concerning post-micturition residual urine volume (PVR) in patients without retention, a 50% reduction seems to have a considerable effect compared with previously published data (PVR reduction: McVary et al.: 38% [29], Mollengarden et al.: 32.3% [7], Darson et al.: 34.9% [6]). The rate of postoperative urinary retention was slightly higher in our study (10.9%) than in the randomized controlled study by McVary et al., who reported urinary retention rates of 4.4% and 5.7% in the two study arms, respectively [5].

Notably, our data showed that the PVol decreased by one-third after therapy. Most studies that evaluated the outcomes of Rezum did not consider its effects on PVol. Mollengarden et al. found a 17% decrease in PVol after Rezum [7]. A recent study by Elterman et al. showed a median decrease in prostate volume of 34% after twelve months [30]. Unlike McVary et al. we were able to demonstrate a significant decrease by 27.5% in PSA levels that from our point of view correlates with the decrease in PVol [5].

In our study, urodynamic measurements were performed before and after treatment in 19/193 patients. In these selected patients, we noted a significant 70% decrease in BOOI. As it concerns only a small number of patients, this finding is certainly of limited value and at best reflects a tendency. However, our results can be useful as a precursor for further randomized controlled prospective studies with larger sample sizes to increase the validity. However, it should be noted that we were able to observe this improvement in BOO in patients who underwent reexamination due to persistent symptoms. As this was a retrospective study, no investigations were conducted on patients who were completely satisfied with the outcome of the treatment. Whether the rate of postoperative deobstruction would have been higher if all patients had undergone repeat urodynamic testing remains speculative. Our study confirms the findings of a previous study that reported a BOOI reduction of 53.8 cmH_2_O [12].

Notably, we treated 35 patients (18.1%) with large prostate volumes ≥ 80 cc. As the number of patients in this subgroup was relatively small, we cannot make any conclusive statements about the effectiveness of the treatment in this specific group. However, recent studies have shown that the effects of Rezum are consistent and do not depend on prostate size [26, 31, 32].

In our clinical experience, Rezum appears to be an effective treatment option, particularly for younger patients with bothersome symptoms and/or those who have experienced failure or side effects of medical treatment for BPE. Additionally, older patients with multiple comorbidities benefit from a shorter operative time associated with Rezum. A recent study reported similar outcomes and low complication rates in patients aged <75 and >75 years [33].

Some authors have suggested performing this procedure under local anesthesia [34, 35]. However, in our study, the operations were carried out under light analgosedation or general anesthesia. In the future, it would be desirable to promote the establishment of regional anesthesia procedures in our clinic. Compared with other studies, hospitalization time was longer; however, this was solely attributed to the German reimbursement system rather than medical factors. In summary, our results suggest that Rezum has a significant effect on urodynamically confirmed BOO. Further studies are necessary to determine which patients benefit most from Rezum.

## Limitations of this study

The major limitations of this study are the retrospective design as well as the high number of patients lost to structural follow-up in our clinic and therefore the small sample size, partly as a result of structural division of the inpatient and outpatient healthcare system in Germany. An additional challenge could be that patients with a good response to therapy were less likely to voluntarily present for outpatient follow-up, especially during the COVID-19 pandemic. Nevertheless, further prospective, large-scale studies are necessary to confirm our findings.

Unfortunately, we could not provide data on BPH medications because they were not assessed systematically. Finally, we were only able to provide urodynamic data for a limited number of patients with urodynamic measurements before and after treatment. As these patients are most likely a negative selection, as described above, positive findings should be even more encouraging.

## Conclusions

This retrospective analysis confirmed that Rezum is a minimally invasive, safe, and effective therapeutic option for patients with BPH-related LUTS. Our data suggest that in addition to the known clinical improvement of symptoms, Rezum can also contribute to a significant improvement in BOO.

## Data Availability

The datasets generated and/or analyzed during the current study are available from the corresponding author on reasonable request.

## References

[1] Gratzke C, Bachmann A, Descazeaud A, Drake MJ, Madersbacher S, Mamoulakis C, et al. EAU guidelines on the assessment of non-neurogenic male lower urinary tract symptoms including benign prostatic obstruction. Eur Urol. 2015;67:1099–109. 25613154 10.1016/j.eururo.2014.12.038

[2] Parsons JK, Dahm P, Köhler TS, Lerner LB, Wilt TJ. Surgical management of lower urinary tract symptoms attributed to benign prostatic hyperplasia: AUA guideline amendment 2020. J Urol. 2020;204:799–804. 32698710 10.1097/JU.0000000000001298

[3] Dixon C, Cedano ER, Mynderse L, Larson T. Transurethral convective water vapor as a treatment for lower urinary tract symptomatology due to benign prostatic hyperplasia using the rezūm® system: Evaluation of acute ablative capabilities in the human prostate. Res Rep Urol. 2015;7:13. 25674555 10.2147/RRU.S74040 PMC4321608

[4] Mynderse LA, Hanson D, Robb RA, Pacik D, Vit V, Varga G, et al. Rezu¯m system water vapor treatment for lower urinary tract symptoms/benign prostatic hyperplasia: validation of convective thermal energy transfer and characterization with magnetic resonance imaging and 3-dimensional renderings. Urology. 2015;86:122–7. 25987496 10.1016/j.urology.2015.03.021

[5] McVary KT, Gittelman MC, Goldberg KA, Patel K, Shore ND, Levin RM, et al. Final 5-year outcomes of the multicenter randomized sham-controlled trial of a water vapor thermal therapy for treatment of moderate to severe lower urinary tract symptoms secondary to benign prostatic hyperplasia. J Urol. 2021;206:715–24. 33872051 10.1097/JU.0000000000001778

[6] Darson MF, Alexander EE, Schiffman ZJ, Lewitton M, Light RA, Sutton MA, et al. Procedural techniques and multicenter postmarket experience using minimally invasive convective radiofrequency thermal therapy with Rezūm system for treatment of lower urinary tract symptoms due to benign prostatic hyperplasia. Res Rep Urol. 2017;9:159–68. 28861405 10.2147/RRU.S143679 PMC5572953

[7] Mollengarden D, Goldberg K, Wong D, Roehrborn C. Convective radiofrequency water vapor thermal therapy for benign prostatic hyperplasia: a single office experience. Prostate Cancer Prostatic Dis. 2018;21:379–85. 29282358 10.1038/s41391-017-0022-9

[8] Johnston MJ, Noureldin M, Abdelmotagly Y, Paramore L, Gehring T, Nedas TG, et al. Rezum water vapour therapy: promising early outcomes from the first UK series. BJU Int. 2020;126:557–8. 32777175 10.1111/bju.15203

[9] Garden EB, Shukla D, Ravivarapu KT, Kaplan SA, Reddy AK, Small AC, et al. Rezum therapy for patients with large prostates (≥80 g): initial clinical experience and postoperative outcomes. World J Urol. 2021;39:3041–8. 33392646 10.1007/s00345-020-03548-7 PMC7779102

[10] Ines M, Babar M, Singh S, Iqbal N, Ciatto M. Real-world evidence with the Rezūm system: a retrospective study and comparative analysis on the efficacy and safety of 12 month outcomes across a broad range of prostate volumes. Prostate. 2021;81:956–70. Sep 1 34254333 10.1002/pros.24191

[11] Campobasso D, Siena G, Chiodini P, Conti E, Franzoso F, Maruzzi D, et al. Composite urinary and sexual outcomes after Rezum: an analysis of predictive factors from an Italian multi-centric study. Prostate Cancer Prostatic Dis. 2023;26:410–4. 36042295 10.1038/s41391-022-00587-6

[12] Martinelli E, Cindolo L, Grossi FS, Kuczyk MA, Siena G, Oelke M. Transurethral water vapor ablation of the prostate with the Rezūm system: urodynamic findings. Neurourol Urodyn. 2023;42:249–55. 36335610 10.1002/nau.25076

[13] Höfner K, Kramer AEJL, Tan HK, Krah H, Jonas U. CHESS classification of bladder-outflow obstruction - a consequence in the discussion of current concepts. World J Urol. 1995;13:59–64. 7539680 10.1007/BF00182667

[14] Schäfer W. Analysis of bladder-outlet function with the linearized passive urethral resistance relation, linPURR, and a disease-specific approach for grading obstruction: from complex to simple. World J Urol. 1995;13:47–58. 7773317 10.1007/BF00182666

[15] Lim CS, Abrams P. The Abrams-Griffiths nomogram. World J Urol. 1995;13:34–9. 7539679 10.1007/BF00182664

[16] Abrams P. Bladder outlet obstruction index, bladder contractility index and bladder voiding efficiency: three simple indices to define bladder voiding function. BJU Int. 1999;84:14–15. 10444116 10.1046/j.1464-410x.1999.00121.x

[17] Oelke M, Rademakers KLJ, van Koeveringe GA. Unravelling detrusor underactivity: development of a bladder outlet resistance—Bladder contractility nomogram for adult male patients with lower urinary tract symptoms. Neurourol Urodyn. 2016;35:980–6. Nov 1 26235823 10.1002/nau.22841

[18] Mitropoulos D, Artibani W, Biyani CS, Bjerggaard Jensen J, Rouprêt M, Truss M. Validation of the clavien–dindo grading system in urology by the European association of urology guidelines Ad hoc panel. Eur Urol Focus. 2018;4:608–13. 28753862 10.1016/j.euf.2017.02.014

[19] Schäfer W, Abrams P, Liao L, Mattiasson A, Pesce F, Spangberg A, et al. Good urodynamic practices: uroflowmetry, filling cystometry, and pressure-flow studies. Neurourol Urodyn. 2002;21:261–74. 11948720 10.1002/nau.10066

[20] Woo H, Gonzalez R. Perspective on the Rezūm® system: a minimally invasive treatment strategy for benign prostatic hyperplasia using convective radiofrequency water vapor thermal therapy. Med Devices. 2017;10:71–80. 10.2147/MDER.S135378 PMC5414627 28490907

[21] Winkler T, von Klot CAJ, Madersbacher S, Kuczyk MA, Wolters M. Rezum water vapor thermal therapy for treatment of lower urinary tract symptoms: a retrospective single-centre analysis from a German high-volume centre. PLoS ONE. 2023;18:e0279883. 36607843 10.1371/journal.pone.0279883 PMC9821484

[22] Chang W, Cheng J, Allaire J, Sievert C, Schloerke B, Xie Y, et al. Shiny: web application framework for R. R package version 1.7. 2.9000, https://shiny.rstudio.com/. Vol. 23, Retrieved February. 2022.

[23] von Klot CAJ, Köpp C, Kuczyk MA, Wolters M. ShinyLUTS—A Shiny web application for structured data management and analysis for patients with lower urinary tract symptoms (LUTS). PLoS ONE. 2023;18:e0292117. 37756331 10.1371/journal.pone.0292117 PMC10530040

[24] Wickham H, Francois R, Henry L, Müller K. Dplyr: a Grammar of data manipulation, 2013. https://github.com/hadley/dplyr. version 0.1.[p 1] 2017.

[25] Wickham H, Chang W, Henry L, Pedersen TL, Takahashi K, Wilke C, et al. Package ‘ggplot2’ version 3.3.0 Create Elegant Data Visualisations Using the Grammar of Graphics. R Journal. 2020.

[26] Bole R, Gopalakrishna A, Kuang R, Alamiri J, Yang DY, Helo S, et al. Comparative postoperative outcomes of Rezūm prostate ablation in patients with large versus small glands. J Endourol. 2020;34:778–81. 32408768 10.1089/end.2020.0177

[27] Dixon C, Cedano ER, Pacik D, Vit V, Varga G, Wagrell L, et al. Two-year results after convective radiofrequency water vapor thermal therapy of symptomatic benign prostatic hyperplasia. Res Rep Urol. 2016;8:207–16. 27921028 10.2147/RRU.S119596 PMC5123707

[28] Cindolo L, Campobasso D, Conti E, Uricchio F, Franzoso F, Maruzzi D, et al. Do patients treated with water vapor therapy and meeting randomized clinical trial criteria have better urinary and sexual outcomes than an unselected cohort? J Endourol. 2023;37:323–9. 36453237 10.1089/end.2022.0637

[29] McVary KT, Rogers T, Roehrborn CG. Rezūm water vapor thermal therapy for lower urinary tract symptoms associated with benign prostatic hyperplasia: 4-year results from randomized controlled study. Urology. 2019;126:171–9. 30677455 10.1016/j.urology.2018.12.041

[30] Elterman D, Bhojani N, Chughtai B, Zorn KC. Change in prostate volume and symptom improvement in men treated With Rezūm water vapor therapy. Urology. 2023;177:142–7. 37076022 10.1016/j.urology.2023.04.008

[31] Elterman D, Bhojani N, Vannabouathong C, Chughtai B, Zorn KC. Rezūm therapy for ≥ 80-mL benign prostatic enlargement: a large, multicentre cohort study. BJU Int. 2022;130:522–7. 35466513 10.1111/bju.15753

[32] Woo H, Levin R, Cantrill C, Zhou S, Neff D, Sutton M, et al. Prospective trial of water vapor thermal therapy for treatment of lower urinary tract symptoms due to benign prostatic hyperplasia in subjects with a large prostate: 6- and 12-month outcomes. Eur Urol Open Sci. 2023;58:64–72. 38152482 10.1016/j.euros.2023.10.006 PMC10751540

[33] Minore A, Morselli S, Franzoso F, Maruzzi D, Varvello F, Toso S, et al. Is water vapor thermal therapy safe and feasible in elderly and frail men? The Italian experience. World J Urol. 2024;42:60. 38280069 10.1007/s00345-023-04762-9

[34] Eredics K, Wehrberger C, Henning A, Sevcenco S, Marszalek M, Rauchenwald M, et al. Rezūm water vapor therapy in multimorbid patients with urinary retention and catheter dependency. Prostate Cancer Prostatic Dis. 2022;25:302–5. 34588631 10.1038/s41391-021-00462-w

[35] Siena G, Sessa F, Cindolo L. Use of a Schelin Catheter for analgesia during Rezum treatment of the prostate. Prostate Cancer Prostatic Dis. 2024;27:147–9. 36639547 10.1038/s41391-023-00644-8 PMC9838278

